# Understanding and Improving the Oil and Water Barrier Performance of a Waterborne Coating on Paperboard

**DOI:** 10.1021/acsapm.2c00937

**Published:** 2022-07-28

**Authors:** Sterre Bakker, Lynn Bosveld, Gerald A. Metselaar, A. Catarina C. Esteves, Albert P. H. J. Schenning

**Affiliations:** † Laboratory of Stimuli-responsive Functional Materials and Devices, Department of Chemical Engineering and Chemistry, Eindhoven University of Technology, P.O. Box 513, 5600 MB Eindhoven, The Netherlands; ‡ BASF Nederland B.V., Innovatielaan 1, 8447 SN Heerenveen, The Netherlands; § Laboratory of Physical Chemistry, Department of Chemical Engineering and Chemistry, Eindhoven University of Technology, P.O. Box 513, 5600 MB Eindhoven, The Netherlands

**Keywords:** paperboard, barrier, waterborne coating, oil, water, aqueous dispersion, alkali-soluble resin

## Abstract

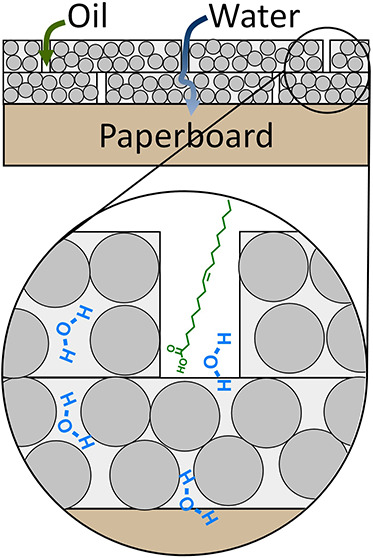

Using paperboard as packaging material is more sustainable than using plastic. To be a viable replacement, however, the barrier properties of paperboard need to be improved. Applying a waterborne barrier coating for both oil and water is an attractive method to improve the barrier performance of paperboard food packaging. However, not much is known about the oil and water barrier properties and penetration pathways of such coatings. Here, an alkali-soluble resin (ASR)-stabilized waterborne emulsion polymer was prepared and applied on untreated paperboard. Its performance as oil and water barrier coating was investigated, and the penetration pathways for both oil and water through the coating are discussed. The presence of surface defects in the coating applied on the paperboard strongly affects both the oil and water barrier properties, but the coating’s morphology and chemical nature only play a major role in the water barrier performance. The optimal barrier performance for oil and water was achieved when adding 5 wt % isopropanol (IPA) to the dispersion and applying two coating layers on paperboard. The IPA improves film formation and reduces the number of surface defects, which is explained by a more favorable spreading coefficient of the coating over the paperboard substrate. These insights will help to improve the oil and water barrier properties of polymer-coated paperboard for more sustainable packaging applications.

## Introduction

Food packaging increased the shelf life and safety of food in such way that we can no longer imagine a life without packaged food. Plastic packaging is widely used for its exceptional barrier properties and durability. However, plastics, having a petrochemical origin, are a substantial source of environmental pollution due to their non-biodegradability. A more sustainable alternative is paperboard packaging since it is a bio-based, nontoxic, recyclable, biodegradable, and inexpensive material.^1−3^ The drawback of using paperboard is its poor barrier properties due to the porous and hydrophilic nature of the natural cellulose-based materials. Therefore, a barrier coating can be applied to protect the paperboard against influences of, for instance, water (vapor), grease, oil, and gases to guarantee the quality of the packaged food. Ideally, these barrier coatings should have excellent barrier properties for both water and oil, minimize environmental pollution, and ensure the easy recyclability of paperboard packages.^1,4,5^ The advantage of using such a barrier coating is that only one step is needed to meet all barrier requirements. The approach for achieving this is to combine both hydrophilic and hydrophobic properties in a single coating to obtain good oil and water barrier performance, respectively. This approach is much more favorable when compared to other strategies that involve chemical modification of the paperboard fibers or blending in of additives and particles. A few cases following the approach of hydrophobic/hydrophilic barrier coatings have been reported,^6−9^ for example, a waterborne coating based on chitosan-*graft*-castor oil copolymer applied on paper where chitosan improved the oil resistance and the grafted castor oil increased the hydrophobicity resulting in good water resistance.^6^ Another example is a waterborne suspension of whey protein concentrate, beeswax, and sucrose applied on paper, which shows a good barrier for both water and oil. In this case, the beeswax was added to increase the hydrophobicity and therefore improve the water barrier properties.^7^ The disadvantages of using bio-based coatings are that these coatings are often sensitive to humidity and processing can be challenging due to thermal instability and crystallization behavior of bio-based polymer materials.^10^

In this research, a waterborne barrier coating is studied for its barrier performance for both oil and water. A polymer dispersion in water stabilized by an alkali-soluble resin (ASR) was prepared by emulsion polymerization and applied on a paperboard substrate to form a water-insoluble coating.^11−15^ ASR-stabilized waterborne coatings can meet the requirements of having both good water and oil barrier performance due to the combined hydrophobic and hydrophilic structure of the ASR, for example, styrene and monomers with carboxylic acid groups, respectively. The ASR is dissolved in alkaline water and acts as stabilizer in an emulsion polymerization process in which a polymer dispersion is formed.^16^ The ASR forms the shell around the polymer particles. This polymer dispersion is then applied on a substrate, and a film is formed, in which the polymer particles and surrounding matrix of the ASR can both be distinguished. Because of the core/shell nature of the polymer particles, the hard ASR shell presumably hinders extensive particle coalescence of the soft cores.^17−21^ In addition, during drying, the partly dissolved ASR precipitates and forms a physical barrier between the soft particles, which is also hindering extensive particle coalescence. Increasing the drying temperature and approaching the *T*_g_ of the ASR improve the particle coalescence.^22−31^ In our previous work, we showed that these ASR-based coatings provide very good water barrier performance when applied on pretreated paperboard.^32^

Herein, we investigate the oil and water barrier performance of ASR-based waterborne coatings on untreated paperboard. Also, the difference in penetration pathways of the oil and water barrier properties and the influence of isopropanol (IPA) addition to the waterborne coating formulation were researched. The oil barrier performance of single coatings with various thicknesses versus double-layer coatings are evaluated visually by using image analysis over time.

## Results and Discussion

**Figure 1:**
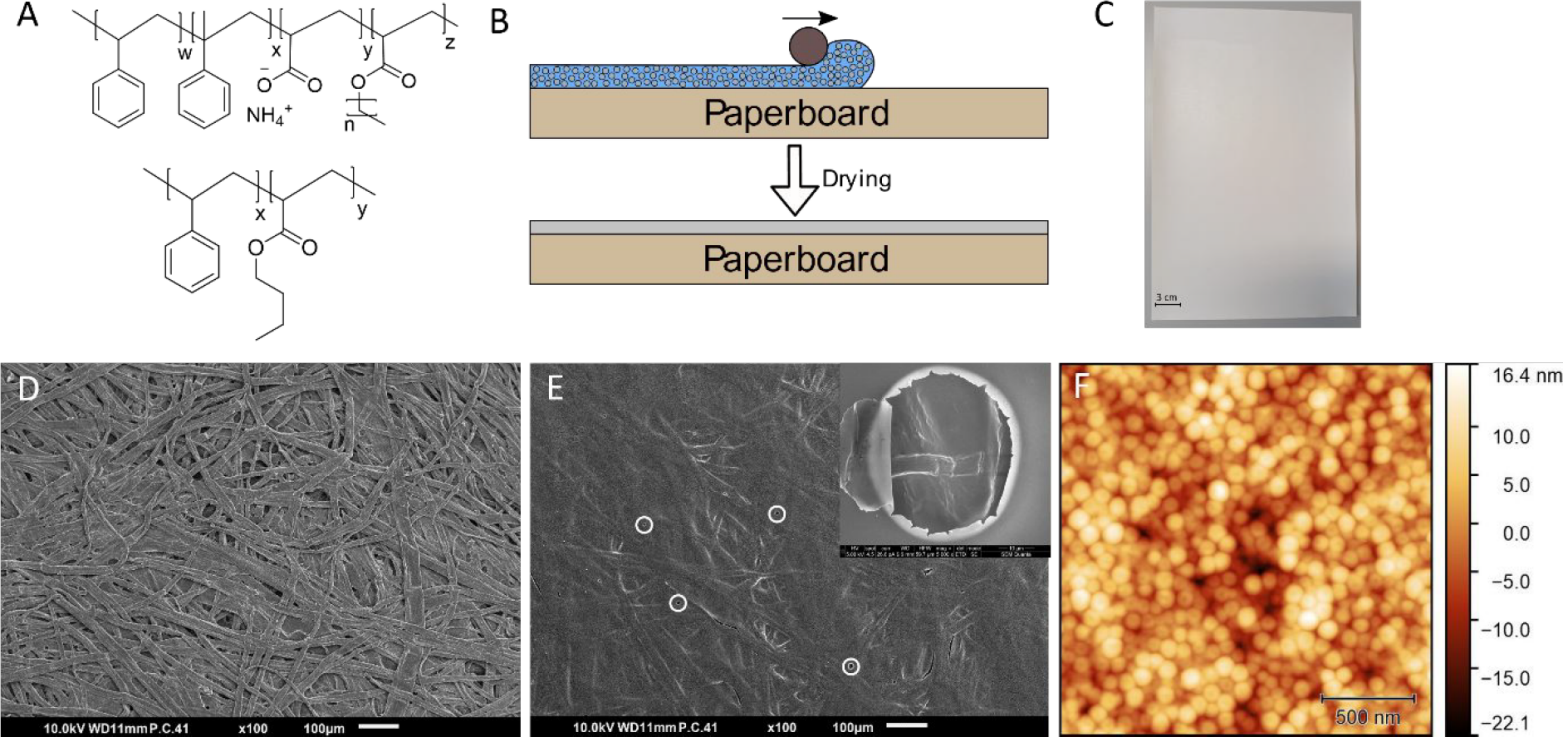
(A) Chemical composition of the waterborne coating showing the ASR and poly(styrene–butyl acrylate) components. (B) Coating formation by bar coating on paperboard and (C) photograph of coating applied on white paperboard. SEM images of the surface of (D) uncoated and (E) coated paperboard; the inset shows the zoom-in of a defect. The defects in (E) are highlighted with white circles. (F) AFM height image of the surface of coated paperboard.

The resin-stabilized dispersion was synthesized by semibatch emulsion polymerization as reported earlier, resulting in poly(styrene–butyl acrylate) polymer particles stabilized by alkali-soluble resin (ASR) in water (Figure 1A).^32^ The undiluted polymer dispersion was directly applied on untreated paperboard by bar coating and dried at 60 °C for 1 h (Figure 1B). This resulted in a transparent and glossy film having a thickness of ca. 6 μm on the white paperboard (Figures 1C and S1). SEM characterization (Figure 1D) showed that the surface of the uncoated paperboard consists of a porous fiber structure. Applying the coating resulted in the coverage of these fibers; however, large defects were visible at the air–coating interface (Figure 1E). Two effects play a role in forming these defects, namely, the rapid evaporation of water and concomitant increasing viscosity of the applied film at the surface. Evaporation of water from inner parts of the coating is hindered by the higher viscosity at the surface where water evaporates faster and a denser film is formed. As a result, this solvent (water) vapor and/or air bubbles are trapped beneath the surface which later breaks through the formed film, resulting in defects called popping.^33^ Independently of these surface defects, a detailed evaluation of the topography of the coating surface, as measured by atomic force microscopy (AFM), showed randomly packed spherical polymer particles (Figure 1F). As discussed in our previous work, these particles originate from the hard shell/soft core particles in the waterborne dispersion.^32^ The hard ASR shell hinders extensive particle coalescence, causing the soft polymer particles to be trapped in an ASR matrix and retaining their initial spherical character.

### Oil Barrier Performance

**Figure 2:**
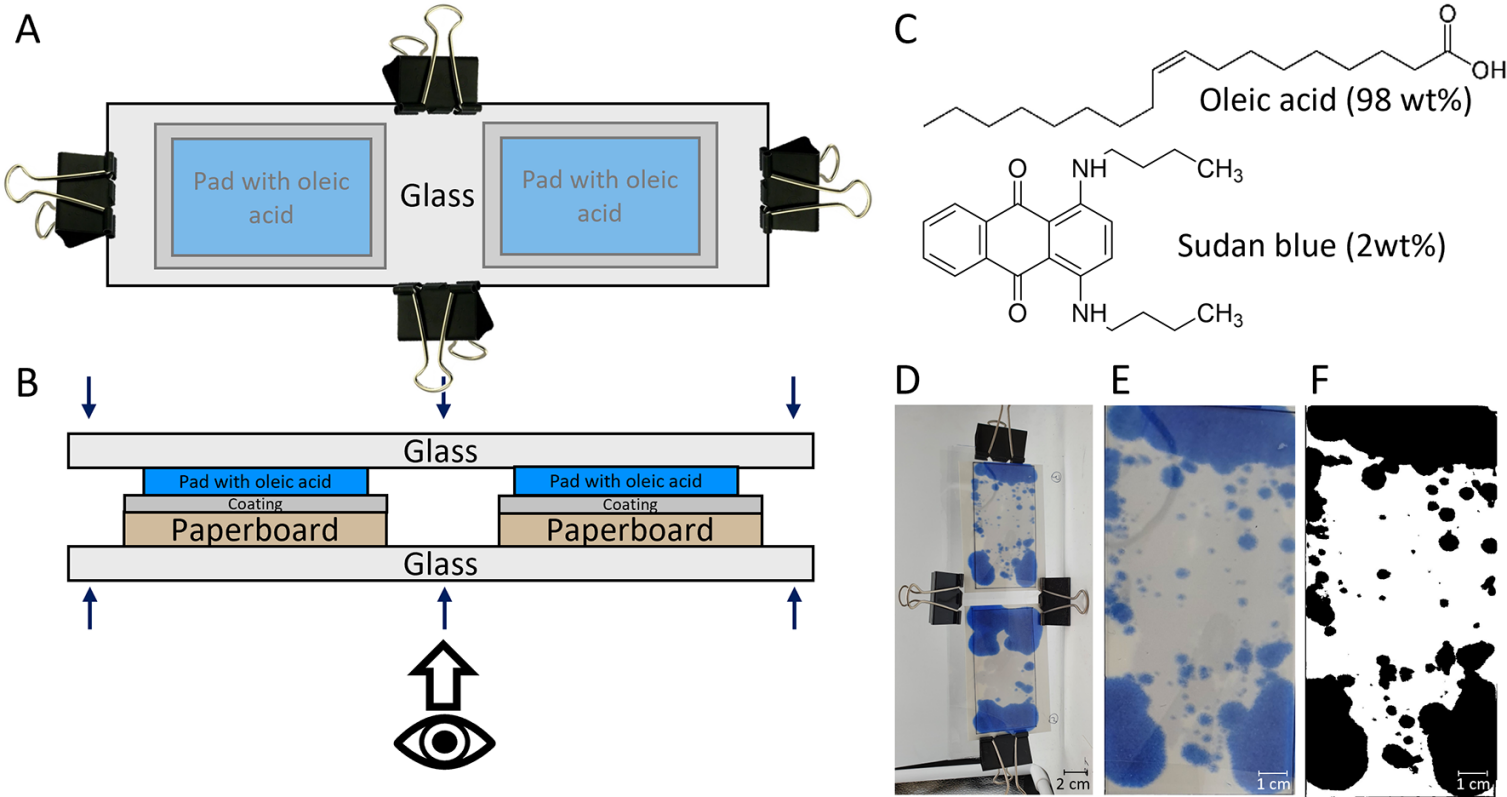
Schematic representation of the oil barrier measurement setup: (A) topview and (B) side view; (C) dyed oil solution used for the oil barrier performance measurements; (D) photograph of the oil barrier setup; the area of interest was (E) subtracted and (F) converted and analyzed.

The oil barrier performance was determined visually by using oleic acid stained with Sudan Blue (Figure 2C).^34^ The oleic acid was distributed over the pad and placed in contact with the coated side of the paperboard, and this was clamped between two glass plates (Figure 2A,B). The oil penetration was determined visually by taking photographs at time intervals of 5, 15, 30, 60, 120, 180, and 240 min from the backside of the coated sample (Figure 2D). The area of interest was subtracted from the photographs, which had the same size and location as the stained pad (Figure 2E). This image was transferred to a black and white pixelated image by using ImageJ software (Figure 2F). The amount of oil penetration was calculated by using eq 1. Besides the oil penetration, also the number of individual oil spots was counted; thus, the area covered with oil was divided by the number of spots to give a general overview of oil penetration behavior.
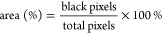
1

**Figure 3:**
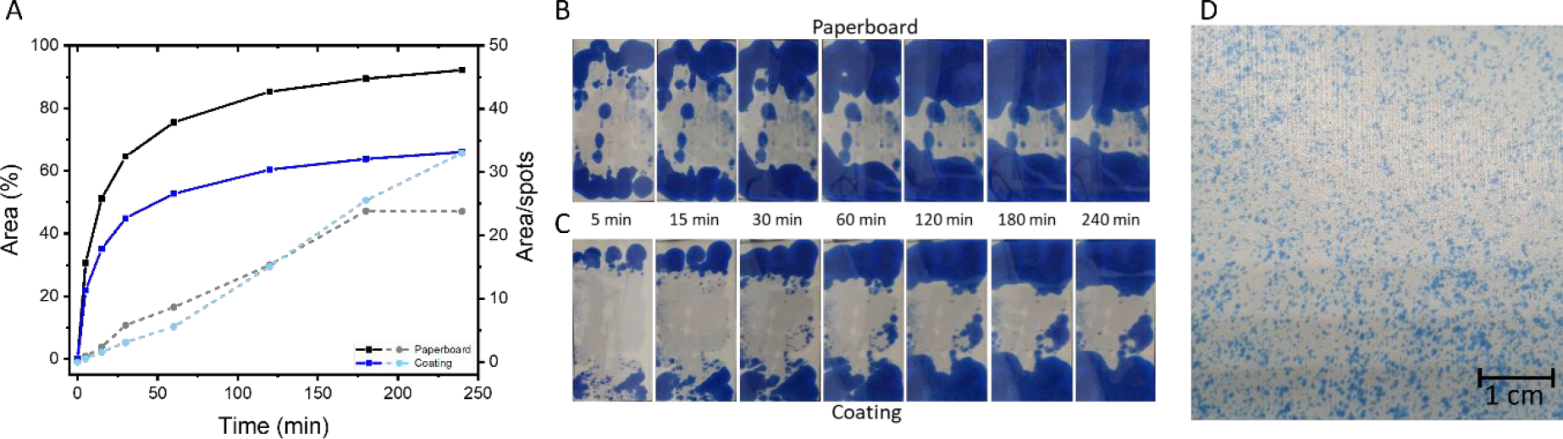
(A) Oil penetration over time plotted for uncoated and coated paperboard (area (%): solid line; area/spots: dashed line). The development of the oil penetration over time. Photographs were taken at different time intervals, and the area of interest was subtracted for (B) uncoated and (C) coated paperboard. (D) Surface defects in the coating were visualized by using stained oleic acid.

Figure 3A shows that 30% of the area was covered with oil within 5 min for uncoated paperboard, which increased to 92% in 4 h. Applying a single layer of resin-stabilized waterborne coating on the paperboard decreased the value to 22% within 5 min and 66% after 4 h. These values indicate poor oil barrier properties. The penetration rate is the highest in the first 30 min, after which it slows down for both uncoated and coated paperboard. Initially, small spots of oil penetrate through the paperboard. These spots then grow and merge, as can be seen by the increasing value for the area divided by the number of spots. Figure 3B,C shows that the oil penetration is mainly located at the top and bottom of the sample. At these locations the pressure between the stained pad and coating is the highest due to the clamping positions, resulting in a higher penetration rate. To exclude the effect of the higher pressure at the clamping positions, the images were divided into three parts, and only the middle part was analyzed (Figure S2). After 4 h, a smaller percentage of the assessed area was covered with oil, indicating less oil penetration. A similar behavior was observed for both the middle and entire area of coated paperboard. Therefore, the higher pressure had only an influence on the amount of oil penetration but not on the penetration rate. This rather poor oil barrier performance of the coated paperboard is ascribed to the presence of a high number of surface defects. These surface defects were visualized by applying an excess of stained oleic acid directly over the coating surface and immediately removing the stained oleic acid from the coating surface with a clean tissue paper (Figure 3D). At the defects, where no coating was present, the blue oleic acid was absorbed by the paperboard, resulting in a blue spot that could no longer be removed during the cleaning step. Indeed, a large number of oil spots were observed, indicating the presence of many surface defects in this coating.

It has been reported that higher drying temperatures improve the film formation of ASR-stabilized waterborne coatings. Particularly, drying temperatures close to the *T*_g_ of the ASR ensure softening of the shell, which is favorable for particle coalescence.^32^ Therefore, the oil barrier performance was tested for coatings dried at room temperature, 60 °C, or 100 °C. Surprisingly, the drying temperature and therewith the coating morphology did not significantly influence the oil barrier performance (Figure S3). Our previous work showed that allowing a sufficiently long equilibration time at ambient conditions is important for the final coating properties. The carboxylic acids of the ASR form an equilibrium with humidity (water vapor) in the air, during which part of the uncharged carboxylic acids convert into the charged carboxylates. A high concentration of carboxylates is unfavorable for the water barrier performance, as the carboxylates are more hydrophilic than carboxylic acids.^32^ The oil barrier performance was determined for 1- and 14-day equilibration time, and it was found that it was independent of the equilibrium time between application and measurement as shown in Figure S4. Hence, these results indicate that, in contrast to the water barrier performance, the oil barrier is not dependent on the chemistry of the coating-substrate system, i.e., on the remaining carboxylate ion concentration. Additional dynamic vapor sorption (DVS) experiments using heptane vapor (Figure S5) showed that the coating itself (free-standing film) hardly absorbs any heptane at 25 °C during a long exposure time (800 min at 80% heptane). This is a good indication that the coating has some inherent good barrier properties against apolar substances, which is the case of oil and grease compounds, and the oleic acid used here to test the oil barrier performance.

### Improving Oil Barrier Performance

**Figure 4:**
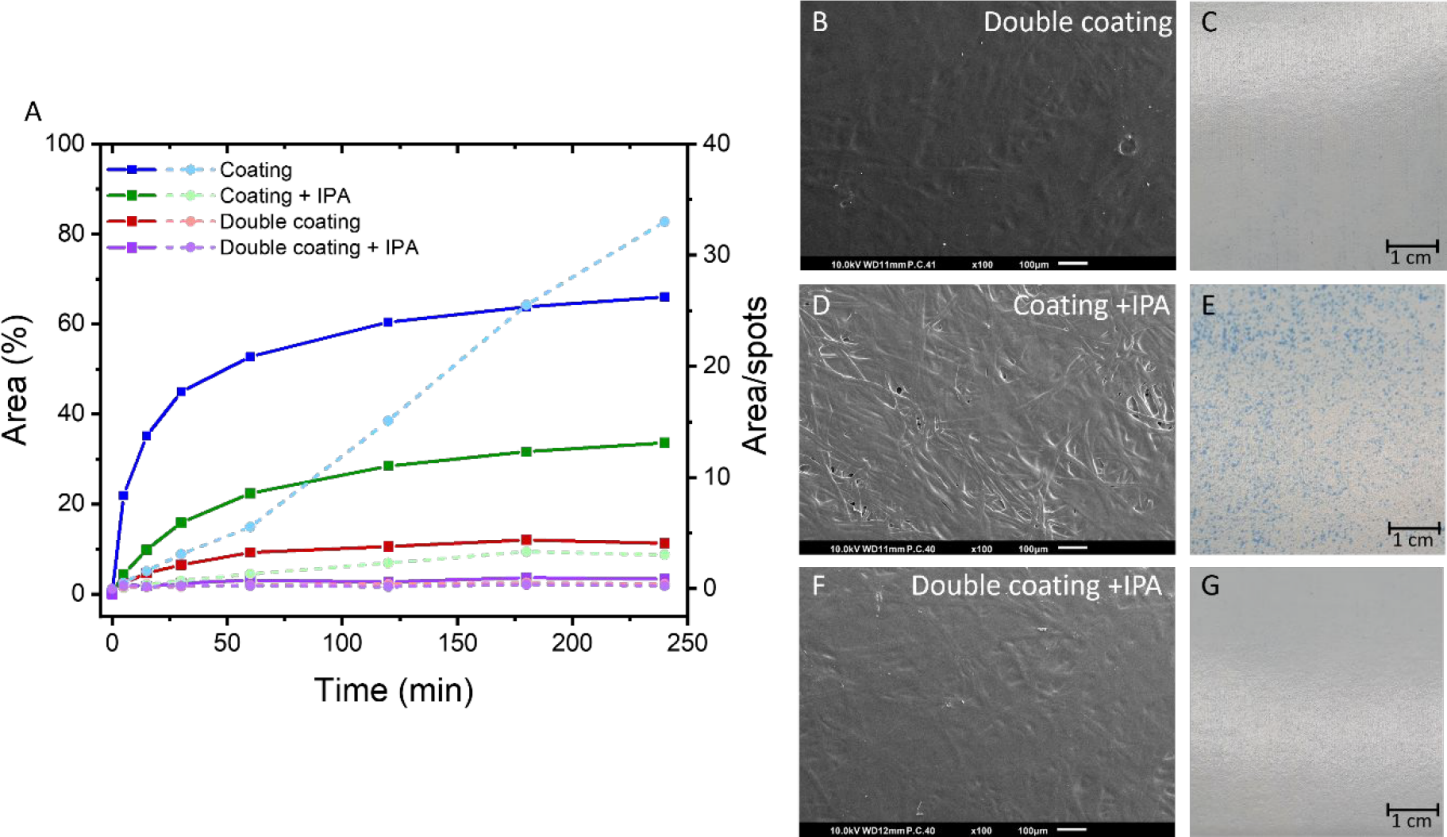
(A) Oil penetration of various coated paperboards (area (%): solid line; area/spots: dashed line). (B) SEM image of the surface of double-coated paperboard and (C) surface defects in double-coated paperboard visualized by using stained oleic acid. (D) SEM image of the surface of coated paperboard with the addition of 5 wt % isopropanol (IPA) and (E) visualization of the surface defects in the coating. (F) SEM image of the surface of double-coated paperboard with the addition of 5 wt % IPA and (G) visualization of the surface defects in the coating.

It was clearly shown above that the oil barrier performance of a single coating layer on untreated paperboard is poor. Therefore, a second coating layer was applied, which doubled the coating thickness to ca. 12 μm (Figure S1). This resulted in a highly improved oil barrier performance (Figure 4A). After 5 min, no oil penetration was detectable, and after 4 h only 11% of the area analyzed was covered with oil. The most likely explanation for this improved performance is that the first applied layer acts as a primer, which makes the porous surface smoother and covers a large part of the substrate pores. The eventual popping defects present after the first coating layer were covered by the second coating layer (Figure 4B,C), which strongly improves the oil barrier performance. Some surface defects were visible in the second coating layer (Figure 4B); however, these are at different locations than the defects of the first layer and have therefore no negative effect on the oil barrier performance. As a comparison for the two-layer coating, also a thicker single-layer coating (ca. 9 μm) was investigated (Figure S1). The thick single-layer coating had even worse oil barrier performance than the 6 μm single-layer coating due to the presence of a high number and larger defects in the coating (Figures S6 and S7). On the basis of these results, the oil barrier performance is shown to be highly dependent on the number of defects rather than on the coating thickness.

To further improve the oil barrier performance, isopropanol (IPA) was added to the polymer dispersion before coating application. IPA was chosen since it is miscible with water has a low vapor pressure, which means that it will easily evaporate at the early stages of film formation. FTIR measurements confirmed the instant evaporation of IPA during drying of the coating (Figure S9). Furthermore, it has been reported that the addition of IPA to aqueous dispersion of polymer binders, i.e., paints or inks, improves the wettability of the porous substrates which may result in better adhesion of the coating and coverage of the paper substrate.^35^ The addition of 5 wt % IPA to the dispersion before application on paperboard resulted in an improved oil barrier for both the single- and double-layer coating (Figure 4A). After 5 min, hardly any oil had penetrated through the coating and paperboard, and after 4 h, only 33% of the area was stained with oil for a single coating layer. Applying two coating layers showed a very good oil barrier since only 3% of the area was colored after 4 h, which is related to the very low number of defects (Figure 4F,G). To shed light on the effect of IPA on the improved oil barrier performance, the surface free energy of IPA:water mixture (5:95 weight ratio) was determined. Water is known to have a surface free energy of 72.7 mN/m, and the surface free energy of 5 wt % IPA in water was determined to be 48.8 mN/m by using pendant drop measurements, which is consistent with values reported in the literature.^36−40^ Note that these surface free energies were determined for pure aqueous solutions without polymer particles because it was not possible to determine the surface free energy of the dispersion due to instantaneous film formation at the dispersion–air interface. The spreading coefficient was also determined following the Owens, Wendt, Rabel, and Kaelble (OWRK) method, which gives information about the tendency of a liquid to wet a solid surface.^41^ A spreading coefficient close to zero means good wetting. The spreading coefficient of water on paperboard was determined to be −60 mN/m, and for the IPA:water mixture on paperboard it was −23 mN/m. Hence, the addition of IPA led to a spreading coefficient closer to zero, which indicates improved wetting compared to water alone. It is assumed that IPA has a similar effect on the wetting of the paperboard by the aqueous ASR-stabilized polymer dispersion. Thus, the introduction of IPA on the aqueous polymer dispersion introduces a nonpolar component to the surface energy of the mixture, which will create a lower interfacial tension and improve the contact and wetting of the paperboard substrate. An improved wetting of the substrate by the presence of IPA can also explain the reduction of popping defects on these coatings. The polymer dispersion will more easily penetrate the pores of the substrate reducing the amount of air/moisture entrapped, in both the substrate and inner layer of the coating, which will then result in less popping defects.^42,43^ We can, however, not rule out the possibility that the presence of IPA could also have other effects that play a role in the film formation such as a plasticizing effect or acting as a coalescence aid.^40,44,45^

### Water Barrier Performance

**Figure 5:**
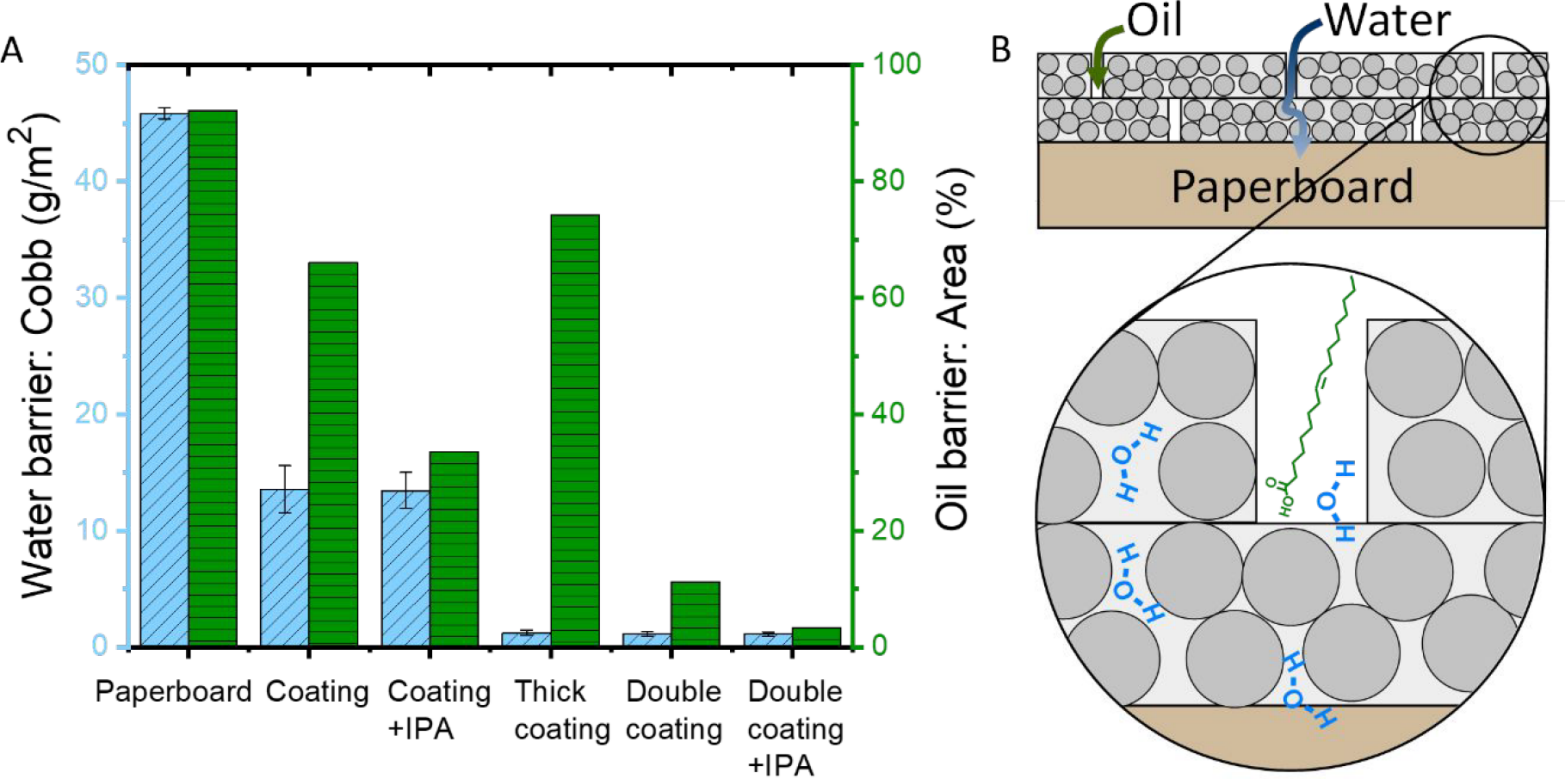
(A) Water barrier performance determined by Cobb and corresponding oil barrier performance by the percentage of the stained area after 4 h of the various coatings applied on paperboard. (B) Proposed mechanism for the oil (green arrow) and water (blue arrow) barrier.

Besides oil barrier properties, also the water barrier performance of the coated substrates was investigated. This was done by measuring the water barrier via the gravimetrical Cobb method. For this method, the difference in mass before and after 10 min of water exposure was determined for uncoated as well as single and double coated paperboard with and without the addition of IPA. The lower the Cobb value, the better the water barrier performance, as less water has been absorbed by the coating and paperboard. Uncoated paperboard has a Cobb value of 46 g/m^2^, which was reduced to 14 g/m^2^ by applying a single coating layer (Figure 5A). Applying two layers further reduced it to 1 g/m^2^, which indicates the best water barrier performance. The addition of 5 wt % IPA to the dispersion prior to coating application did not affect the water barrier, in contrast to the effect observed for the oil barrier. This corresponds with our previous findings, which show that the water barrier performance is highly dependent on carboxylate concentration of the ASR in the coating rather than on the film formation and structural properties.^32^ This is further supported by the results of the thick single-layer coating of ca. 9 μm, which has a Cobb value of 1 g/m^2^ (Figure 5A). This value is comparable with the Cobb results of double-layer coating. Therefore, the water barrier performance improves with increasing coating thickness applied on paperboard. It should be noted that all coatings applied showed the same particle structure, independently of the thickness, number of layers, or IPA presence/absence (as shown by the AFM analysis in Figure S10).

The combined results from the oil and water barrier performance provide evidence that the barrier properties for water and oil are due to different mechanisms. The uptake of oils into paper-based materials occurs mostly via penetration into the pores of the substrate. Water and water solutions are mostly taken up by fiber sorption although can also be partially absorbed via the pores.^43^ In the case of the current coating, there is also a direct chemical interaction between water (vapor) and the functional groups of the ASR-based coating, i.e., carboxylate ions.^32^ Hence, the oil barrier is improved by efficiently embedding the substrate pores with polymer and thus minimizing the defects through which the oil could penetrate more easily into the substrate. The water barrier properties are, however, determined by the chemistry of the coating layers and in this case by the presence of the ASR-based matrix surrounding the polymer particles containing residual carboxylate ions (as illustrated in Figure 5B). Accordingly, the presence of IPA in the aqueous polymer solution has a strong impact on the oil barrier by reducing the porosity and defects, while the application of a thicker or double layer has a strong impact on the water barrier performance. The combination of a double layer and IPA gives the best barrier for both oil and water.

## Conclusion

An ASR-stabilized polymer dispersion was synthesized by emulsion polymerization and applied on untreated paperboard to form a waterborne barrier coating. A single-layer coating with a thickness of ca. 6 μm showed poor oil barrier performance due to the presence of surface defects. The oil barrier performance was strongly improved by applying a second coating layer which minimizes these defects. The best oil barrier performance was achieved by adding 5 wt % IPA to the dispersion and applying two coating layers on paperboard. The spreading coefficient and coating penetration into the porous substrate were more favorable after the addition of IPA, which resulted in improved film formation with hardly any popping defects. The best water barrier performance was also found for double coating layers. However, the addition of IPA did not influence the water barrier performance. For both the oil and water barrier performance, it is important to minimize the number of defects in the film and to have low permeability, solubility and diffusion for both oil and water substances. However, oil and water permeation follow different wetting and coating–substrate penetration paths. Oil penetrates mainly through defects and pores in the coating and substrate, which means that an excellent oil barrier can be made if the defects and porosity are minimized. For water, another effect also plays a role, namely the chemical structure of the coating. The carboxylate concentration in the ASR-based matrix in the coating applied here is dependent on the humidity and determines permeability of water in the coating. Therefore, the water barrier performance was mainly improved by applying more coating material on the paperboard, via application of either a thicker single- or double-layer coating. The results of this work demonstrate the mechanisms behind the barrier performance of the ASR-based coatings applied on paperboard and show the potential of using resin-stabilized waterborne coating as a barrier against both water and oil.

## Experimental Section

### Materials

For the emulsion polymerization, styrene (S, ≥99%) and *n*-butyl acrylate (BA, ≥99%) were purchased from Sigma-Aldrich. The alkali-soluble resin (ASR) solution and ammonium persulfate were used as received from BASF. More information about the ASR was reported previously (Figure 1A).^32^ For the oil barrier analysis, oleic acid (≥90%), Sudan Blue II (1,4-bis(butylamino)anthracene-9,10-dione, ≥98%), and isopropanol (IPA, ≥99.5%) were purchased from Sigma-Aldrich. All chemicals were used as supplied. The paperboard substrate was kindly provided by Storaenso, type “Ensocard”. This is an uncoated bleached board having a thickness of 215 μm (170 g/m^2^).

### Coating Preparation

The polymer dispersion was prepared via semibatch emulsion polymerization performed in a reactor by using mechanical stirring. Initially, ASR aqueous solution (19.50 g) and water (6.00 g) were added to the reactor and heated to 84 °C under an argon atmosphere. The thermal initiator (0.15 g in water (0.60 g)) was added to the reactor. After 3 min, the styrene (7.25 g) and butyl acrylate (7.25 g) were added to the reaction mixture over 2 h. Subsequently, water (2.50 g) was flushed through the feed tube, and the reaction was kept at *T* = 84 °C for an additional hour. More water (3.75 g) was added, and the reaction mixture was cooled to room temperature in ambient conditions and filtered (50 μm mesh size). More details are described in our previous work.^32^ Coatings were prepared by applying ca. 1.5 mL of the polymer dispersion on the paperboard substrate (18 × 30 cm^2^) and making a “draw-down” using the bar coater (RK control coaster, speed level 8, 12, or 24 μm wet deposit wire bar). The coatings were dried at 60 °C for 1 h in a ventilated oven. For the double layered coatings, a second layer was applied following the same procedure, after drying the first layer at 60 °C for 1 h. For the coatings with the addition of 5 wt % IPA, the dispersion was added to the IPA and mixed by hand. After mixing the dispersion was immediately coated on paperboard and dried at 60 °C for 1 h. After drying, the coatings were stored under ambient conditions for further characterization.

### Coating Characterization

#### Scanning Electron Microscopy (SEM)

SEM images were taken of the surface and cross section of the coatings applied on paperboard. The cross section was prepared by cryogenic breaking of the sample in liquid nitrogen. All samples were covered with a conducting layer by sputter coating with gold (30 s at 30 mA), and images were taken with SEM (JEOL 7800F) in secondary electron mode. The thickness of the coating layer was estimated by analyzing the cross sections of the samples in the SEM images.

#### Atomic Force Microscopy (AFM)

Topography images of the coating surface were made with AFM in AC mode (tapping mode). AFM imaging was performed with Cypher ES Environmental AFM equipped with a closed cell and a heating–cooling stage. The silicon probe (model AC160TSA) was manufactured by Olympus and purchased from Asylum Research. The probe has a 7 nm tip radius and a 14 μm tip height and operates with a spring constant of *k* = 26 N/m and a frequency of 300 kHz.

#### Visualizing Coating Defects

The coating defects were visualized by distributing stained oleic acid (dyed with 2 wt % Sudan Blue) over the coating surface with tissue paper. Immediately after that (maximum 30 s), the excess of blue oleic acid was removed with tissue paper. The defects were colored blue after this procedure, and photographs were taken of the coating–air interface.

#### Surface Free Energy Measurements

The pendant and sessile drop measurements were performed on a Dataphysics OCA30 contact angle goniometer with the OGA20 software to determine the spreading coefficient. The pendant drop measurement was used to determine the surface free energy of water, IPA, and IPA:water mixture (5:95 weight ratio). The surface free energy was estimated from an average of 10 droplets of 2 μL. Sessile drop measurements were used to determine the static contact angle, from which the polar and dispersive parts of the surface free energy of paperboard, double-coated paperboard, water, and IPA:water mixture were derived. The liquids used for the measurement were water (Milli-Q), diiodomethane (Sigma-Aldrich, 99%), and ethylene glycol (Sigma-Aldrich, 99%) on (coated) paperboard. Also, the contact angle of water and IPA:water on PTFE plate (Sysmex) was measured. The static contact angle was calculated from the average of the left and right angles of 10 droplets of 2 μL. The OWRK theory was used for the calculations.^41^

#### Barrier Performance

The oil barrier performance was determined qualitatively. The coated substrates were dried for 1 h at 60 °C, followed by an equilibration step of 1 day under ambient conditions. The coated samples of 11 × 7 cm^2^ were covered at the coating side with a 10 × 6 cm^2^ glass fiber pad (CEM Square Sample Pads). 0.8 g of oleic acid (dyed with 2 wt % Sudan Blue) was distributed over the pad. Two coated samples covered with pads were clamped between two glass plates (25 × 9.5 cm^2^) by using four clamps. At defined time intervals, 5, 15, 30, 60, 120, 180, and 240 min, a photograph was taken of the uncoated “backside”, i.e., substrate/glass side. The oil penetration was determined by data analysis using the ImageJ software^46^ (https://imagej.nih.gov/). For the data analysis, the photographs were cropped and rotated in such way that the area of interest matched with the size and location of the oil penetrated pad. The percentage of the stained area was calculated, and the average of four samples was taken. Also, the number of stained spots was calculated, and the percentage of the area was divided by the number of spots. The (liquid) water barrier properties were determined by using the Cobb method as described in our previous work.^32^

## Supporting Information Available

SEM images of the cross sections of the coatings on paperboard; oil penetration behavior of the middle part of the samples; influence of drying temperature on oil barrier performance; influence of equilibration time on oil barrier performance; DVS measurement of heptane vapor; oil barrier performance of a single thick coating; visualizing surface defects; photographs of oil penetration behavior over time; AFM height images of coating surfaces (PDF)
