# Supplementary material for: Understanding and Improving the Oil and Water Barrier Performance of a Waterborne Coating on Paperboard
Source: ACS Appl Polym Mater. 2022 Jul 28;4(8):6148–55. doi: 10.1021/acsapm.2c00937 (PMC9379909; doi:10.1021/acsapm.2c00937)
Supplement: Supplementary file 1 — ap2c00937_si_001.pdf [file ap2c00937_si_001.pdf]

Supporting information

Understanding and improving the oil and water barrier performance of a waterborne coating on paperboard

Sterre Bakker[a], Lynn Bosveld[a], Gerald A. Metselaar[b], A. Catarina C. Esteves[c], Albert P.H.J. Schenning[a]**

[a] Laboratory of Stimuli-responsive Functional Materials and Devices, Department of Chemical Engineering and Chemistry, Eindhoven University of Technology, P.O. Box 513, 5600 MB Eindhoven, The Netherlands

[b] BASF Nederland B.V., Innovatielaan 1, 8447 SN Heerenveen, The Netherlands

[c] Laboratory of Physical Chemistry, Department of Chemical Engineering and Chemistry, Eindhoven University of Technology, P.O. Box 513, 5600 MB Eindhoven, The Netherlands

* Email: gerald.metselaar@basf.com / a.p.h.j.schenning@tue.nl

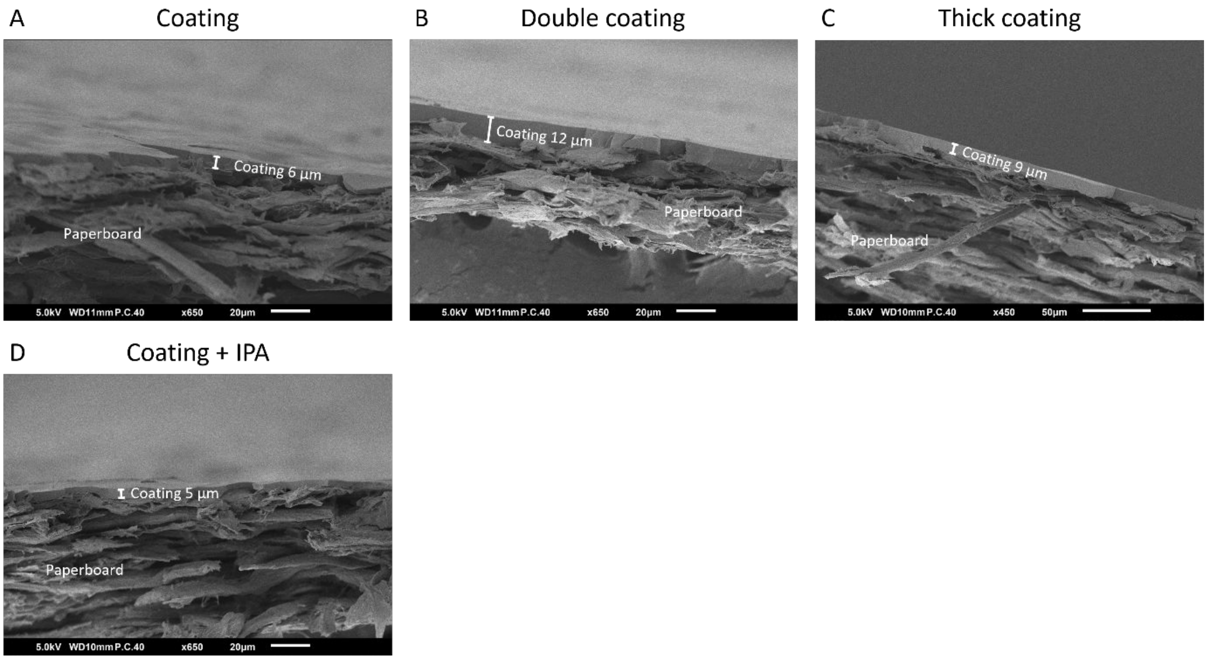

Figure S1. SEM images of the cross-sections of various coatings applied on paperboard showing the layer thickness.

The oil barrier performance was determined for the middle part of the images in order to exclude the effect of the higher-pressure area between pad and coating at the clamping positions. The images used for the analysis were divided into three parts (**Figure S2**B), the oil penetration in the middle part was shown in **Figure S2**A.

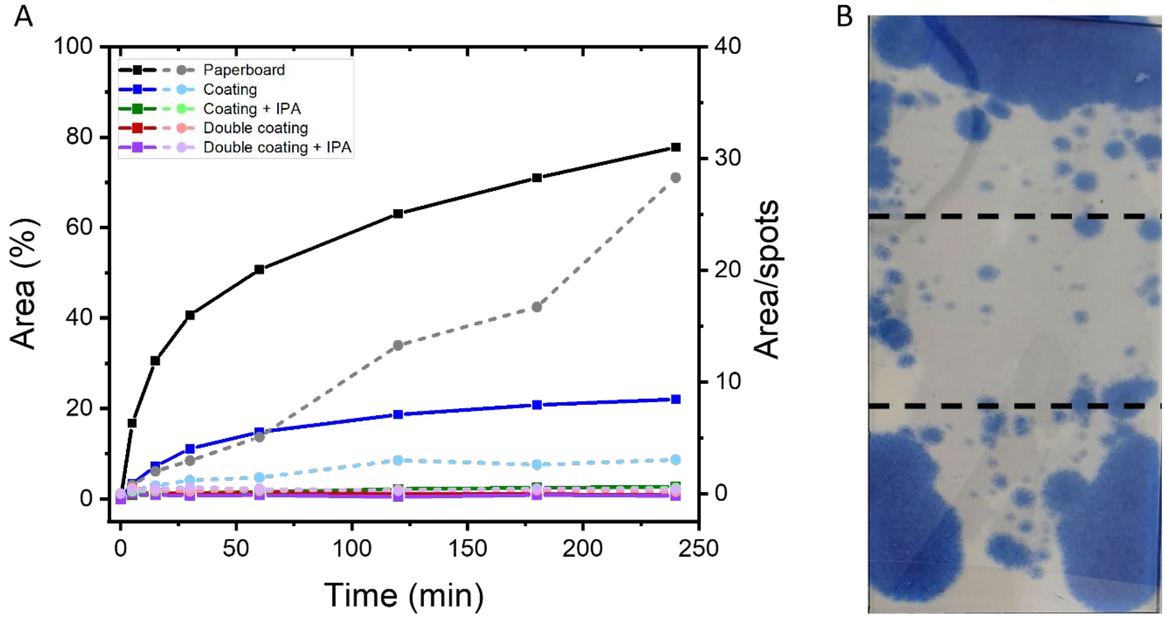

Figure S2. A) The oil penetration of the middle part of the coated paperboard (area (%) – solid line, area/spots – dashed line). B) Photograph of the oil penetration divided in three parts. The middle part is analyzed in A).

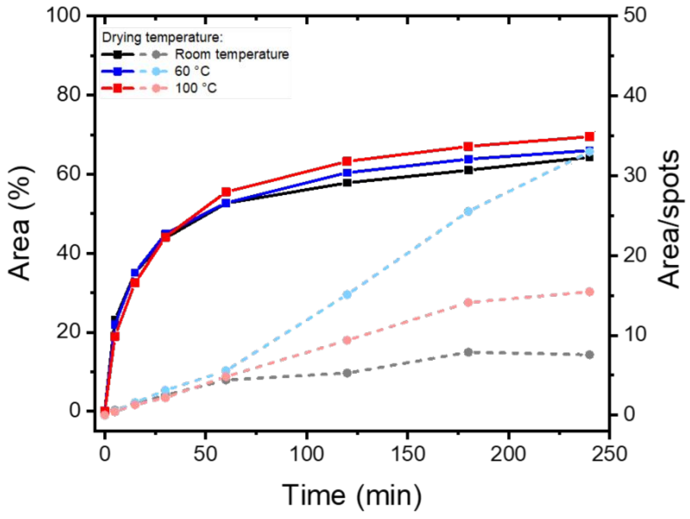

Figure S3. The oil penetration of coated paperboard dried at room temperature, 60 ˚C and 100 ˚C (area (%) – solid line, area/spots – dashed line).

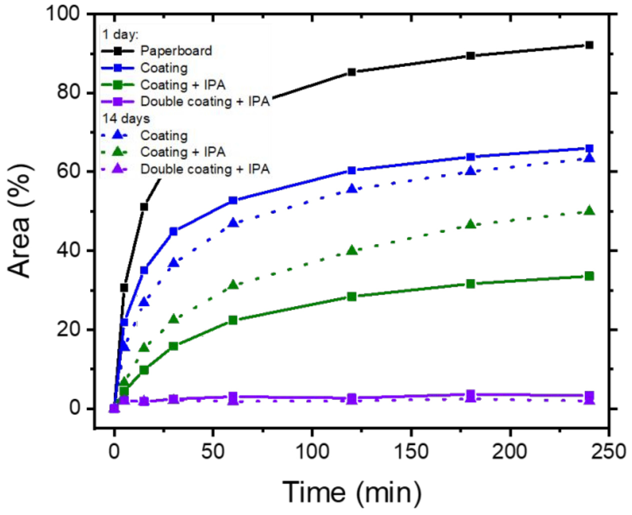

Figure S4. The oil penetration of coated paperboard determined after 1- or 14-days after coating application (area (%) – solid line, area/spots – dashed line).

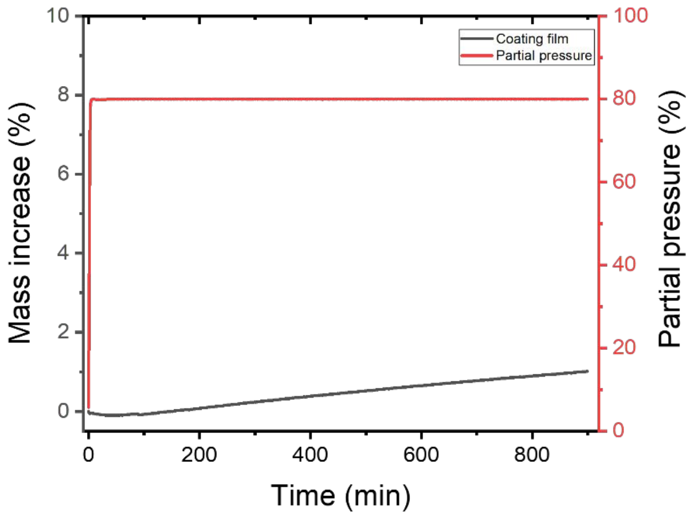

Figure S5. The weight increase of a free-standing film of the coating at 80% partial pressure of heptane vapor at 25 ˚C measured by dynamic vapor sorption.

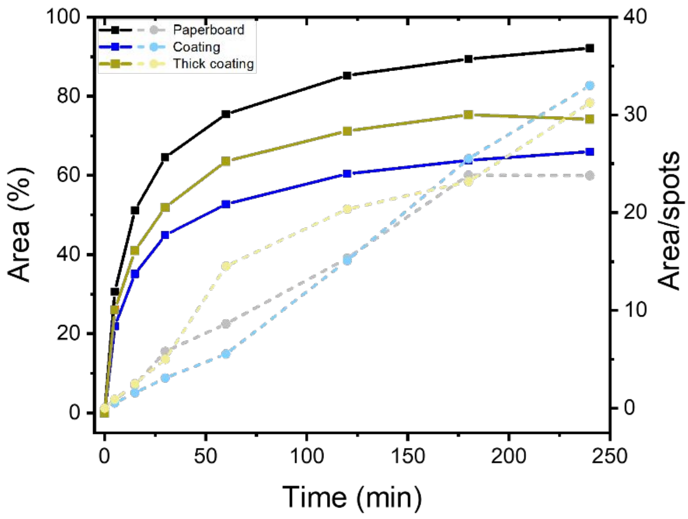

Figure S6. The oil penetration of coated paperboard in comparison with a thicker coating (area (%) – solid line, area/spots – dashed line).

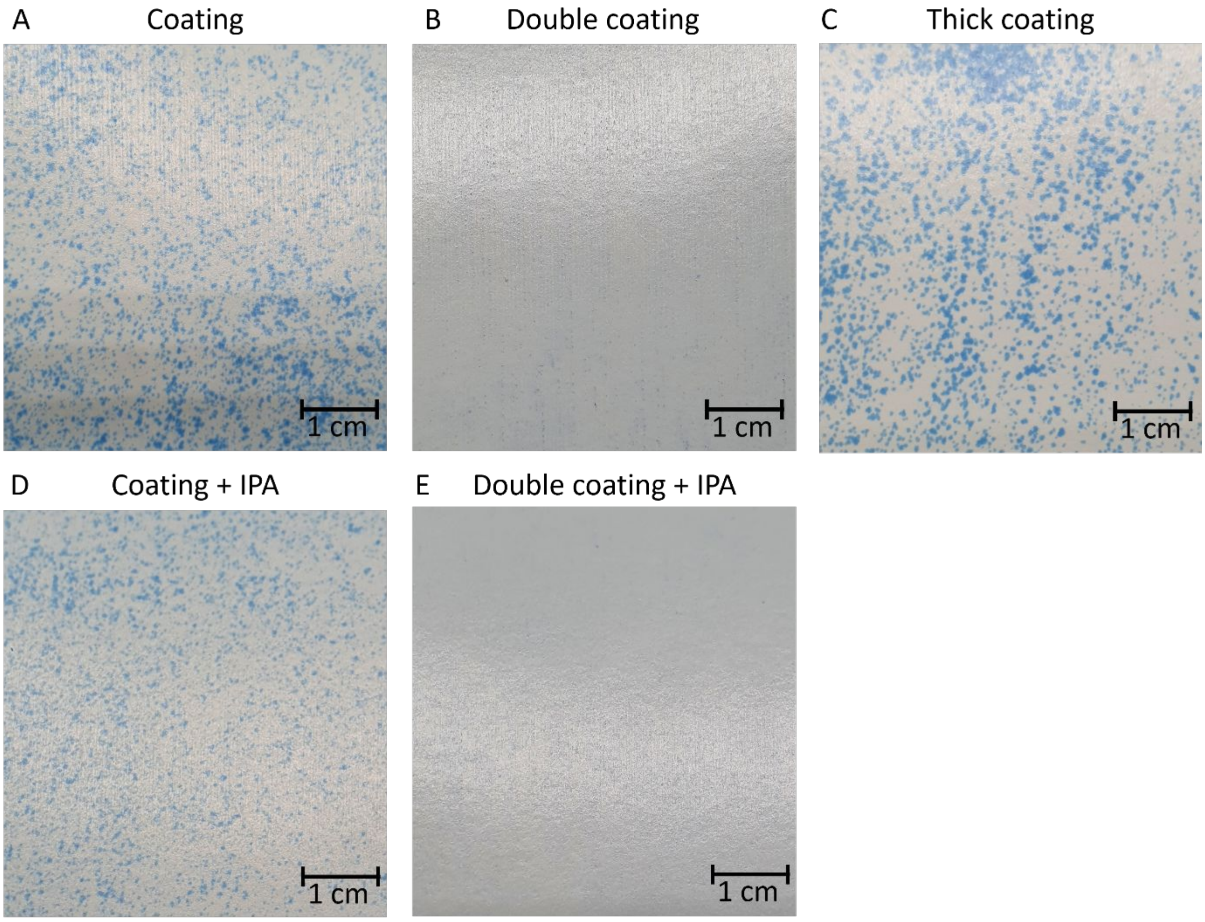

Figure S7. The defects in various coatings were visualized by making photographs of the coatings directly embedded with stained oleic acid.

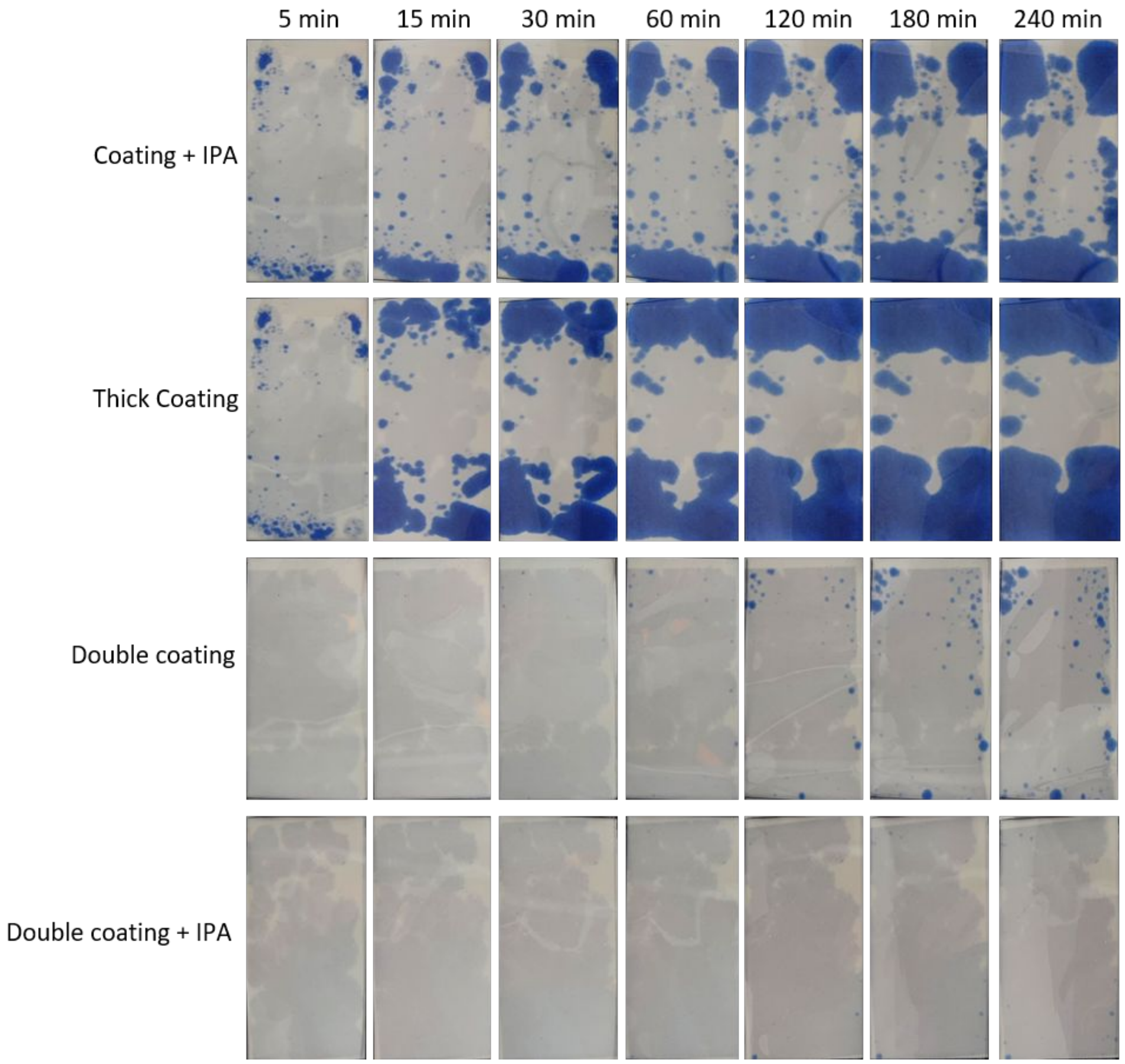

Figure S8. The development of the oil penetration over time. Photographs were taken at different time intervals and the area of interest was subtracted.

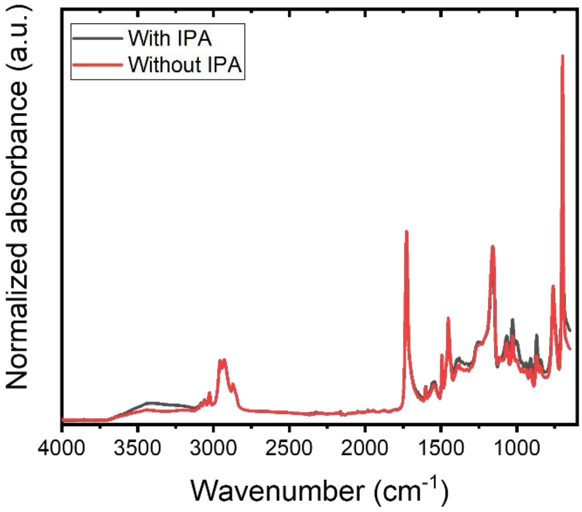

Figure S9. FTIR measurement of the coating with and without the addition of IPA measured after 45 minutes after application on paperboard and dried at room temperature.

The AFM height images show different behavior for the single and double coating layer (**Figure S10**A and B). For a single layer, during drying water evaporates both via the coating-air and coating-paperboard interface since water easily penetrates in the paperboard. In contrast to a single layer coating, for a double layer coating, water evaporates only via the coating-air interface. The surface of the dried double-layered coating appeared to be different compared to a single layer coating measured with AFM, instead of clearly distinguishable particles present, indentations were observed. Also, the height difference of the surface has decreased resulting in a smoother surface.

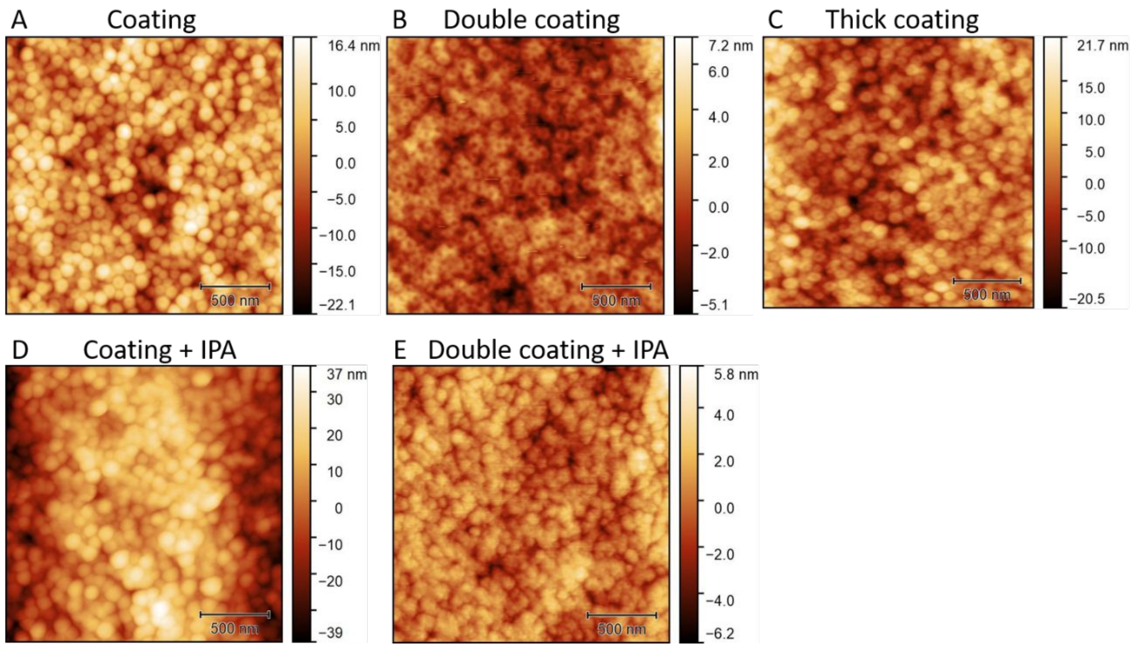

Figure S10. AFM height images of the coating surface applied on paperboard for A), single, B) double and C) thick single coated paperboard. Also, D) single and E) double coated with the addition of 5 wt% isopropanol.
